# New models for energy beam machining enable accurate generation of free forms

**DOI:** 10.1126/sciadv.1701201

**Published:** 2017-09-22

**Authors:** Dragos Axinte, John Billingham, Aitor Bilbao Guillerna

**Affiliations:** 1 Faculty of Engineering, University of Nottingham, Nottingham, UK.; 2 School of Mathematical Sciences, University of Nottingham, Nottingham, UK.

## Abstract

We demonstrate that, despite differences in their nature, many energy beam controlled-depth machining processes (for example, waterjet, pulsed laser, focused ion beam) can be modeled using the same mathematical framework—a partial differential evolution equation that requires only simple calibrations to capture the physics of each process. The inverse problem can be solved efficiently through the numerical solution of the adjoint problem and leads to beam paths that generate prescribed three-dimensional features with minimal error. The viability of this modeling approach has been demonstrated by generating accurate free-form surfaces using three processes that operate at very different length scales and with different physical principles for material removal: waterjet, pulsed laser, and focused ion beam machining. Our approach can be used to accurately machine materials that are hard to process by other means for scalable applications in a wide variety of industries.

## Teaser

A generic mathematical model determines the beam paths for various energy beam machining processes to generate free-form surfaces.

## INTRODUCTION

Energy beam (EB) processes, such as abrasive waterjet (AWJ), pulsed laser ablation (PLA), and focused ion beam (FIB), can be used for controlled-depth machining (material removal) of difficult-to-process materials. This enables the generation of complex free-form surfaces for various applications ranging from medical and microelectromechanical systems to aerospace and defense applications. These EB machining methods provide a set of complementary capabilities:

(1) Length scale (minimum beam diameter): AWJ, macro/meso (>120 μm); PLA, meso/pseudomicro (>5 μm); FIB, micro/nano (>10 nm);

(2) Productivity (material removal rate): AWJ, high (about 3000 mm^3^/min); PLA, medium (0.08 × 10^7^ to 80 × 10^7^ μm^3^/s); FIB, low/very low (0.02 × 10^−2^ to 3 × 10^−2^ μm^3^/s);

(3) Versatility: AWJ, any material; PLA, dependent on the laser absorption coefficient of the material; FIB, needs vacuum;

(4) Surface quality (average absolute height deviations): AWJ, rough [surface roughness (Ra) > 3.6 μm]; PLA, fine (Ra > 0.6 μm); FIB, ultrafine (Ra << 0.6 μm).

**Fig. 1:**
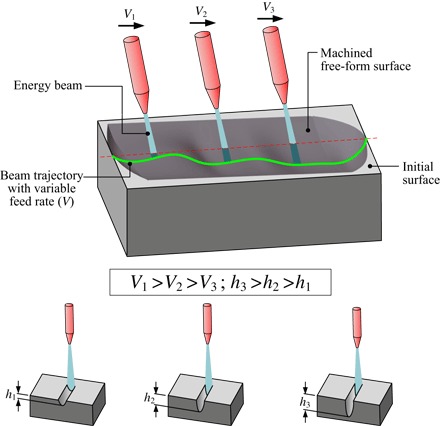
Generic representation of EB controlled-depth machining, variation of the footprint with beam exposure time, and its relative position to the target surface.

In each of these processes, the result of the interaction between the EB and the target surface is a machined footprint whose shape and depth are dependent on energy density and exposure time. When the EB moves in a straight line, the footprint takes the form of a trench that could be of variable depth amplitude with the variation of the beam feed speed, *v*, as suggested in Fig. 1. This can be the result of the continuous action of the EB (AWJ) or of a sequence of overlapping pulses (PLA and FIB) upon the target surface.

Different EB processes use different mechanisms to remove material: mechanical erosion (AWJ), melting and vaporization (PLA), and energy/momentum transfer (FIB). In previous studies, these processes have, therefore, been treated separately from both an experimental and a modeling point of view. In particular, some attempts have been made to model the generation of both single and superposed footprints using physics-based (*1*–*3*) and numerical models (*4*–*6*). These approaches usually involve strong simplifying assumptions and difficult calibration procedures, which leads to long computation times, making them impossible to use in practice for the control of machine tools that can generate complex free forms. Moreover, because these models attempt to capture the detailed physics of each specific removal process, they are not generic, that is, they do not lead to methods that are applicable for a wide range of EB processes in a variety of setups and applications.

In contrast, we have developed a simpler modeling approach that can predict the geometry of the machined footprint for, theoretically, any EB machining process (*7*, *8*). To account for the specific material removal mechanisms by a particular EB process, we performed a set of simple experimental calibrations and identified specific removal rate functions. This allows us to unify the modeling of these processes into a common mathematical framework based on a partial differential evolution equation for the workpiece surface. This equation has a straightforward structure that respects the basic physics of the process, but is simple enough that it can be accurately calibrated using a few initial experiments. Once this has been carried out, single and superposed footprint profiles can be determined for any kinematic EB parameters, that is, path, exposure time along the path, and angle of incidence of the EB. This is the direct problem in EB machining. If the path of the EB is selected on the basis of either the intuition and experience of the end user (craftsmanship) or trial and error, then there can be a large discrepancy between the actual machined surface and the free-form surface that is the required outcome of the EB process.

What is needed here is an algorithm for determining the kinematics of the EB that leads to the required free-form surface. This is the inverse problem in EB machining. If a complex, free-form shape is to be generated, the motion of the beam may need to be carried out repeatedly, removing the material in successive layers, depending on the desired aspect ratio of the free form, while maintaining the original stand-off distance with each successive layer.

Despite the importance of the inverse problem in the generation of free-form surfaces using time-dependent material removal processes, very few investigations on this topic have been reported. Some approaches simply vary the exposure time of the beam on each pixel of the required surface (*9*, *10*); this is simply the leading order approximation to the necessary strategy when the radius of the beam is small compared to the size of the feature that is being etched. However, this does not account for either of the nonlinear effects, the detailed shape of the footprint, nor the effect of the overlapping beam paths. Although it is a plausible starting strategy, particularly for FIB, it is not sufficient for other EB processes or even, in all situations, for FIB. In addition, a Fourier convolution approach to the linearized version of this problem, which does not explicitly take the path of the beam into account, has been studied for abrasive waterjet micromachining, fluid jet polishing, and ion beam figuring in previous studies (*11*–*14*). If the features that need to be machined are comparable to the size of the beam, a more sophisticated approach is needed.

Some reports on the inverse problem for other time-dependent processes include the following: electrochemical machining (*15*), where the tool/electrode works in tangential mode to envelope the required surface, and electrodischarge machining (*16*), where the electrode copies the geometry of the final surface so that a solution of the inverse problem is not required. We recently reported on a solution of the inverse problem in AWJ, working in the linear erosion regime to minimize errors in the generation of simple two-dimensional shapes (*17*).

Our research aims to present a unified method of modeling EB machining that allows us to solve the inverse problem, so that highly accurate free forms can be generated independently of the physics that governs the material removal process. Our approach is simple and efficient and requires only modest computing power to produce the required beam paths and exposure times. We have validated this approach using three different EB machining processes: AWJ, PLA, and FIB.

## RESULTS AND DISCUSSION

The basis of our mathematical model of EB processes is that the boundary of the workpiece evolves as a function of exposure time under the action of the beam. In particular, only the part of the surface that is beneath the beam changes at any instant, and the only explicit spatial dependence of the rate of material removal is given by a removal rate function, *E*(*r*), which depends on the distance from the center of the beam, *r*, alone. We also assume that the physics of the removal process can be decomposed into a set of multiplicative functions, which variously characterize the slope dependence, depth dependence, and beam velocity (EB exposure time) dependence of the rate at which the material is removed. The number and form of these functions vary between processes, but for each process, there is a simple calibration procedure (*8*, *18*).

**Fig. 2:**
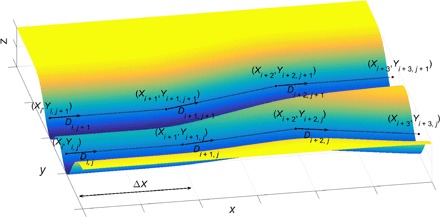
Notation used in parameterizing the beam paths.

We work in a Cartesian coordinate system (*x*, *y*, *z*), with the axis of the beam parallel to the *z* axis (Fig. 2). We will assume that the axis of the beam retains this orientation during the whole machining process. We also assume that the workpiece has an initially flat boundary, given by *z* = *Z*(*x*, *y*, *t*), with *Z*(*x*, *y*, 0) = 0. The evolution equation is

$$\frac{\partial Z}{\partial t}=-E(r(t;u))f_{1}(\nabla Z)f_{2}(|V|)f_{3}(Z)$$

where the path of the center of the beam projected onto the (*x*,*y*) plane is **x** = **X**(*t*;**u**), radial position in the beam is *r* = |**x** − **X**(*t*;**u**)|, and $V\equiv \frac{dX}{dt}$ is the velocity of the beam. The function *f*_1_(∇*Z*) models the dependence of the rate of removal on the slope of the evolving surface, *f*_2_(|**V**|) the dependence on beam speed, and *f*_3_(*Z*) the dependence on machined depth, which captures enough the physics of the processes to give an accurate model. The vector of the control parameters, **u**, specifies the path of the beam (as described below).

We briefly summarize how the functions *f*_1_, *f*_2_, and *f*_3_ are calibrated for each of the three EB processes we have studied. For more details, see the studies of Billingham *et al*. (*7*, *8*).

(1) For AWJ, *f*_2_ = *f*_3_ = 1, only the angle of incidence on the surface is found to nonlinearly affect the process, and the calibration is in two stages. First, the removal rate function, *E*(*r*), is determined from measurements of a straight, shallow trench machined with constant feedspeed, for which *f*_1_ ≈ 1. Under this approximation, the model is linear, and *E*(*r*) can be directly related to the profile across the trench (averaged along the trench to minimize the effect of process noise) through a simple integral. Second, the function *f*_1_(∇*Z*) is determined by machining a straight trench along which the beam speed increases linearly. A quadratic function of the angle of incidence is found to give excellent results.

(2) For PLA, *f*_1_ = *f*_3_ = 1, only the feedspeed is found to nonlinearly affect the process, and the calibration is again in two stages. First, the removal rate function, *E*(*r*), is calibrated in the same manner as for AWJ. Second, the function *f*_2_(|**V**|) is determined by machining a straight trench along which the beam speed increases linearly. For PLA, a linear function of the exposure time is the appropriate functional form.

(3) For FIB, *f*_2_ = 1, both angle of incidence and machined depth, but not beam speed, affect the process. The functional form of *f*_1_(∇*Z*) is well known for FIB (*19*, *20*) and is characterized by two parameters. The function *f*_3_(*Z*) is introduced to account for the way FIB merely damages the surface when the beam speed is large, which appears as a skin effect in the results. The function *f*_3_(*Z*) is chosen to be an exponential that tends to one as *Z* → −∞ (away from the skin), which accounts for this effect, and introduces two additional parameters. Because of this skin effect, the simple procedure that allows *E*(*r*) to be calibrated in AWJ and PLA does not work. However, *E*(*r*) is found experimentally to be close to Gaussian and can therefore be characterized by two parameters. The model parameters can be easily calibrated by machining straight trenches at several beam speeds and measuring the averaged profile across each trench.

Although it is natural to write the evolution equation, with time, *t*, as the independent variable, it is more convenient instead to use arc length, *s*, measured along the beam path. Because *ds*/*dt* = |**V**|, we can write

$$\frac{\partial Z}{\partial s}=-D(s;u)E(r(s;u))f_{1}(\nabla Z)f_{2}(D^{-1})f_{3}(Z)$$

where *D* ≡|**V**|^− 1^ is the exposure time.

For the given beam path parameters, **u**, the forward problem is to integrate the evolution equation forward as the beam moves along its path until *s* = *S*(**u**), where *S*(**u**) is the total arclength of the beam path, and to determine the final etched surface, *Z*(*x*, *y*, *S*;**u**).

For a given required final etched surface, *Z*_*T*_(*x*, *y*), the inverse problem is to find beam path control parameters, **u**, such that *Z*(*x*, *y*, *S*(**u**);**u**) = *Z*_*T*_(*x*, *y*). Partial differential equation–constrained inverse problems like this, where there are finitely many parameters, **u**, and an infinite dimensional target, *Z*_*T*_(*x*, *y*), need to be formulated as an optimization problem. We define the cost function, *J*(**u**)≡‖*Z*(*x*, *y*, *S*(**u**);**u**) − *Z*_*T*_(*x*, *y*)‖^2^, and seek to minimize it over the space of possible control parameters, **u**.

**Fig. 3:**
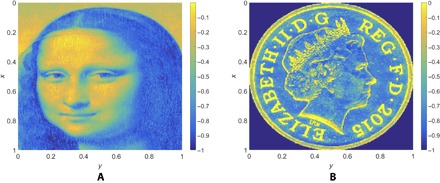
Complex free-form targets used in the experimental trials. The Mona Lisa (**A**) is relatively smooth compared to the British Penny (**B**), which has many sharp edges. The heights of the targets have been normalized here.

For a complex free-form surface (we will use the Mona Lisa and the British penny; Fig. 3), it is likely that the path of the beam that optimally solves the inverse problem is itself complex. However, practical constraints imposed by machine dynamics mean that beam paths with significant high-frequency components cannot be used (*17*). One approach to this problem is to use simple raster paths (that is, parallel beam movements with constant overlapping). For AWJ, paths more complex than this are almost impossible to control because of the complexity and inertial mass of the machine, but for PLA and FIB, the control and dynamic characteristics of the machines are such that more complex paths can be used. We have chosen to use close to raster (small deviations from parallel) paths to demonstrate our solution of the inverse problem because they are a good compromise between complexity and machinability.

On each of the *N*_p_ passes, there are *N*_u_ control points, through which the beam passes in a piecewise linear manner. At each of these points, (*X*_*i*,*j*_, *Y*_*i*,*j*_), the exposure time is *D*_*i*,*j*_. This exposure time is also linearly interpolated between the control points. This fully specifies the beam path. The distance between consecutive points is chosen to be constant in the *x* direction, so that

$$X_{i,j}=X_{i-1,j}+\Delta X\;\text{for}\;1<i\leq N_{u}-1$$

The beam path parameter vector, **u**, is therefore composed of the exposure time at each control point and (unless straight raster paths are used) the *y* coordinate of each control point, so that

$$u=(Y_{0,0},D_{0,0},Y_{1,0},D_{1,0},Y_{2,0},D_{2,0,}\ldots ,Y_{N_{u}-1,N_{p}-1},D_{N_{u}-1,N_{p}-1})$$

The first subscript, 1 ≤ *i* ≤ *N*_u_, denotes the *i*th control point for each raster pass, and the second subscript, 1 ≤ *j* ≤ *N*_p_, indicates the *j*th pass, so there are 2*N*_p_*N*_u_ control parameters in the most general case.

The forward problem is

$$\frac{\partial Z}{\partial s}=-D(s;u)E(r(s;u))f_{1}(\nabla Z)f_{2}(D)f_{3}(Z)$$

subject to *Z*(*x*, *y*, 0;**u**) = 0, for 0 ≤ *s* ≤ *S*(**u**). For a given **u**, and hence a given beam path and exposure time as a function of arc length, a simple, central finite difference scheme with explicit Euler arc-length stepping and a uniform Cartesian grid is sufficient to accurately compute *Z*(*x*, *y*, *S*(**u**);**u**), the final etched surface, and hence the cost function

$$J(u)\equiv {\left||Z(x,y,S(u);u)-Z_{T}(xy)|\right|}^{2}$$

The inverse problem, namely, to find a set of control parameters, u*, such that

$$J(u^{*})\leq J(u)\forall u\in U$$

where *U* is the set of possible control parameter vectors, could be tackled using a wide range of different optimization algorithms. A key point is that we know that a simple pixel-by-pixel approach with straight raster paths and exposure time proportional to the required depth of removal gives a final etched profile that is reasonably close to the target surface. This means that we have a good initial estimate of **u**, so that a simple gradient-based approach is able to locate a local minimum of *J*(**u**), which is in good agreement with the target surface, although we cannot guarantee that this is a global minimum.

To implement gradient-based optimization, we need an efficient way to calculate the gradient matrix, ∂*J*/∂**u**. Because there are typically several thousand control parameters in **u**, the obvious, finite difference approach is prohibitively expensive. Instead, we solve the discrete adjoint to the finite difference solver for the forward problem to efficiently evaluate the gradient. The uniform-grid, explicit Euler finite difference approach is simple enough that we can calculate the adjoint finite difference scheme by hand. This consists of another evolution problem that must be integrated backward along the beam path. This calculation is of comparable computational complexity to the calculation of *J*(**u**) in the forward problem and gives us a very efficient means of calculating the gradient at each step of the optimization.

We have used this methodology to generate free forms on various materials using AWJ, PLA, and FIB as EB machining processes (see the Materials and Methods for more details). We will illustrate our results using complex free forms, namely, the Mona Lisa (a smooth surface) (Fig. 3A) and the British Penny coin (a surface with various sharp edges with different orientations) (Fig. 3B).

For each surface, we show results with straight raster beam paths, and to demonstrate that we can obtain significantly better results for surfaces with sharp edges, we also used nonstraight raster paths for the Penny coin. Note also that for AWJ, we were able to use only straight paths due to practical constraints imposed by machine dynamics, so we only present the results for the Mona Lisa.

**Fig. 4:**
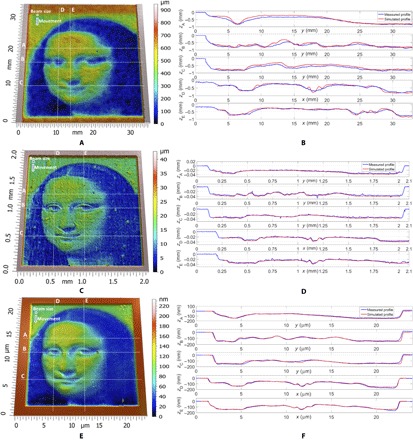
The results of experimental trials using the Mona Lisa, a relatively smooth surface, as the target. These were generated using AWJ (**A** and **B**), PLA (**C** and **D**) and FIB (**E** and **F**). The full surface is shown in each case along with various cross sections through the surface. Agreement between the measured and simulated surfaces is excellent in each case.

Figure 4 shows the Mona Lisa, a typical smooth surface, generated by AWJ (Fig. 4A), PLA (Fig. 4B), and FIB (Fig. 4C) using straight raster paths. The noise inherent in AWJ machining, due to the complex multiphase turbulent fluid flow in the jet and its interactions with the target surface as well as the dynamics of the machine, leads to a significantly less accurate free-form generation with less high-frequency content (small-scale features). In addition, deviations from the required profiles on different cross sections (A, B, C, D, and E) are presented for each process in Fig. 4 (D to F).

Note that the nature of the deviations depends on the orientation of the cross section relative to the raster path. This effect can be observed more on the cross sections A, B, and C of the AWJ machined surface (Fig. 4A) that present higher deviations from the simulated profile. This is caused by the lateral step-over of the beam and the interaction of adjacent trenches, which is more prone to secondary effects that are less well captured by the model. In contrast, for the cross sections D and E, the errors are significantly smaller because they are in the direction of motion of the beam. Figure 4 (C and D) shows that the target machined using PLA is somewhat closer in detail to the required surface than that machined using either AWJ or FIB (see Fig. 4, E and F). This is not due to the error in the machined surfaces themselves, but rather the fact that our model for FIB is inherently nonlinear owing to the skin effect, which means that our algorithm produces simulated surfaces that are significantly less accurate for FIB than for PLA.

**Fig. 5:**
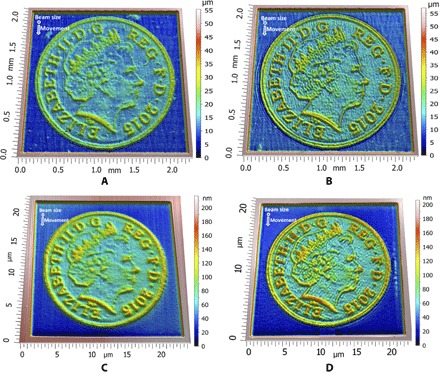
The results of experimental trials using the British Penny, a surface with many sharp edges, as the target surface. These were generated using PLA (**A** and **B**) and FIB (**C** and **D**), with either straight (A and C) or nonstraight (B and D) paths. The dynamics of the bulky AWJ machine preclude the use of nonstraight paths for machining. In each case, nonstraight paths result in a more accurate free-form.

**Fig. 6:**
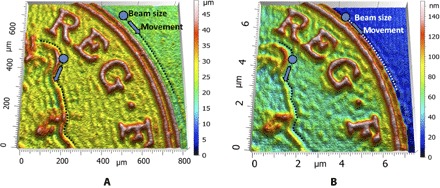
Details of a surface with many sharp edges generated by nonstraight beam paths using PLA (**A**) and FIB (**B**). In each case, note that the beam follows the sharp edges. As a consequence of this, the use of nonstraight passes improves the accuracy of the machining mainly in the neighbourhood of the sharp edges.

Figure 5 shows the British Penny, a typical surface with sharp edges at different orientations, generated by PLA (Fig. 5A) and FIB (Fig. 5C) using straight raster paths and Fig. 5B and Fig. 5D show results using nonstraight raster paths. It is clear that the nonstraight paths are able to more accurately capture the sharpness of the various edges, which demonstrates the utility of our approach. Details of the surfaces generated by nonstraight passes are presented in Fig. 6, where it can be observed that the beam follows the edges of the free form, thus resulting in a better definition of the surface.

## CONCLUSIONS

We have developed a simple generic modeling approach and algorithms for the inverse problem to generate free-form surfaces using different EB machining processes and various workpiece target materials. This modeling approach is able to embed the physics of the diverse range of material removal mechanisms encountered in EB processing using simple experimental calibration. The accuracy of the approach has been demonstrated by low average relative errors (AWJ, 10 to 20%; PLA, 6 to 8%; FIB, 4 to 6%) from the required surfaces. Being a time-dependent modeling framework, our approach is sensitive to the constraints of the machine dynamics that could limit the system response to sudden changes of the feed speed required to generate the free forms; the limitations imposed by the machine dynamics have been put in evidence by generating with AWJ only on smooth surfaces (for example, Mona Lisa) rather than sharp-edged surfaces, which requires sharp changes of the EB feed speed. Hence, our modeling framework needs to be accompanied by appropriate characterization and modeling of the dynamics of the EB machining system. Although here only piecewise linear EB paths have been investigated, future work could be extended to understand the influence of more complex paths (for example, circular and spiral) on the accuracy of the machined free forms.

## MATERIALS AND METHODS

The proposed modeling approach and the efficient solution for determining the inverse solution were validated on three EB processes/machines characterized by different material removal mechanisms, working principles, and dynamics:

(1) Waterjet machining (AWJ) as a continuous macro (beam diameter, 500 μm) attrition-based material removal process. A Microwaterjet 3-axis F4 type (Waterjet AG), with jet positioning accuracy <0.003 mm, equipped with an orifice 0.18 mm in diameter and a focussing tube 0.5 mm in diameter operating at 3500-bar (KMT streamline SL-V100D) pump pressure was used. Using a constant nozzle-to-surface stand-off distance of 3 mm, the jet feed speeds were varied, in accordance with the solution of the inverse problem, between 200 and 600 mm/min. Considering the ability of the process to machine difficult-to-cut materials, Ti6Al4V, an alloy extensively used in aerospace and medical industries, was used as the target workpiece for AWJ.

(2) PLA as a discontinuous meso (beam diameter, 45 μm) melting/vaporization-based material removal process. A pulsed (1 to 35 kHz) SPI-G3 HM fiber laser using an Aerotech AGV-10HP galvanometer was used to manipulate the beam, with the feed speed varying between 4 and 25 mm/s as per the solution of the inverse problem, on two axes. An *f*-θ lens (100-mm focal length) was used to focus the beam on a four-axis Aerotech ACS-150-135 machining table on which we set a flat graphite (POCO AF-5) target material. Using this setup, a beam with an average diameter of 0.045 mm (ellipticity, 0.956) and a measured (Thorlabs PM100D) power of 18.8 W was obtained.

(3) FIB as a micro (nominal beam diameter of 100 nm) momentum-based material removal process was performed using an FEI Helios NanoLab 600 system with a Ga^+^ LMIS (liquid metal ion source) operated with a beam energy of 30 keV and current of 6.5 nA. The FIB chamber pressure was maintained in the order of 10^−6^ mbar during the irradiation. The single crystalline boron p-doped Si substrate with resistivity of 11 to 12 Ω·cm was cleaned in an ultrasonic bath using acetone, isopropanol and deionized water for 10 min and dried with nitrogen gas. To minimize redeposition in the experimental tests, the maximum aspect ratio of the machined structure was kept below 1.

For the free forms generated by AWJ and PLA, a Bruker GT-I white light interferometer (pixel size of 197 nm) was used, whereas the FIB free form was measured with an atomic force microscope using a Bruker Icon Dimension in tapping mode. As an initial free-form surface to demonstrate our models, we chose the Mona Lisa for its various gradients, which was scaled accordingly: AWJ (30 × 30 × 0.8 mm^3^) (Fig. 4A); PLA (1.865 × 1.865 × 0.04 mm^3^) (Fig. 4C); FIB (20 × 20 × 0.175 μm^3^) (Fig. 4E). This scaled free form was used to demonstrate the accuracy of the solution of the inverse problem with straight beam paths parallel to the *y* axis.

To further demonstrate the accuracy of the solution of the inverse problem using nonstraight beam paths, we generated a free form with sharp gradients, namely, the British one penny coin, using PLA (Fig. 5B) and FIB (Fig. 5D), with details presented in Fig. 6, A and B, respectively. This free form was not generated by AWJ because the machining head has high inertia, which is not be able to respond to the fast commands needed to generate the nonstraight paths.
